# Author Correction: Structures of a deltacoronavirus spike protein bound to porcine and human receptors

**DOI:** 10.1038/s41467-023-40128-w

**Published:** 2023-07-20

**Authors:** Weiwei Ji, Qi Peng, Xueqiong Fang, Zehou Li, Yaxin Li, Cunfa Xu, Shuqing Zhao, Jizong Li, Rong Chen, Guoxiang Mo, Zhanyong Wei, Ying Xu, Bin Li, Shuijun Zhang

**Affiliations:** 1 College of Life Sciences, Nanjing Agricultural University, Nanjing, 210095 China; 2 Institute of Veterinary Medicine, Jiangsu Academy of Agricultural Sciences, Key Laboratory of Veterinary Biological Engineering and Technology, Ministry of Agriculture, Nanjing, 210014 China; 3 Jiangsu Key Laboratory for Food Quality and Safety-State Key Laboratory Cultivation Base of Ministry of Science and Technology, Nanjing, 210014 China; 4 Jiangsu Coinnovation Center for Prevention and Control of Important Animal Infectious Diseases and Zoonoses, Yangzhou University, Yangzhou, 225000 China; 5 Central Laboratory of Jiangsu Academy of Agricultural Sciences, Nanjing, 210014 China; 6 College of Veterinary Medicine, Henan Agricultural University, Zhengzhou, 450046 China

**Keywords:** Viral transmission, X-ray crystallography

Correction to: *Nature Communications* 10.1038/s41467-022-29062-5, published online 18 March 2022

In the original version of the acknowledgements section, some of the funding sources were omitted or misattributed. This has been corrected in the PDF and HTML version of the Article.

