# Supplementary material for: Structures of a deltacoronavirus spike protein bound to porcine and human receptors
Source: Nat Commun. 2022 Mar 18;13:1467. doi: 10.1038/s41467-022-29062-5 (PMC8933513; doi:10.1038/s41467-022-29062-5)
Supplement: Supplementary file 1 — Supplementary Information [file 41467_2022_29062_MOESM1_ESM.pdf]

Supplementary information for

Structures of a deltacoronavirus spike protein bound to porcine and human receptors

Weiwei Ji, Qi Peng, Xueqiong Fang, Zehou Li, Yaxin Li, Cunfa Xu, Shuqing Zhao, Jizong Li, Rong Chen, Guoxiang Mo, Zhanyong Wei, Ying Xu, Bin Li, Shuijun Zhang

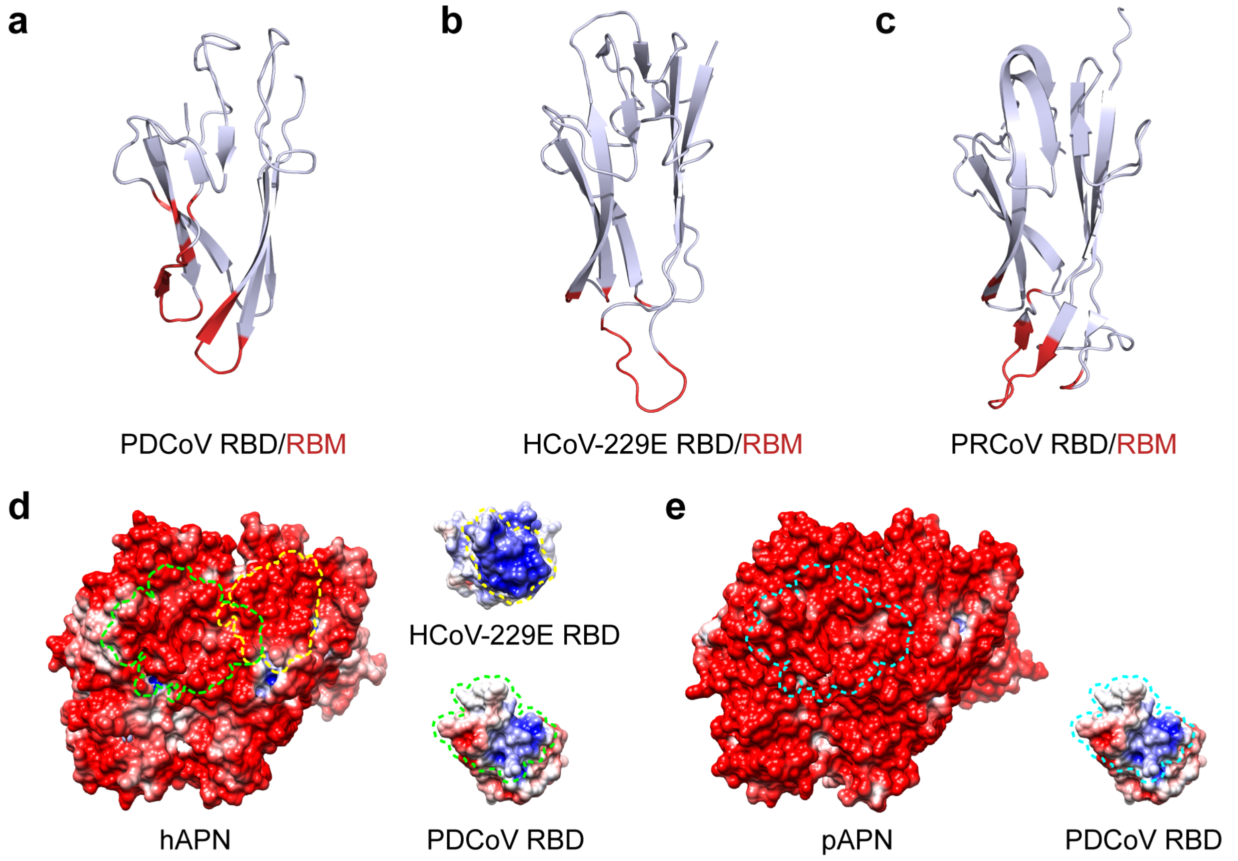

Supplementary Fig. 1 Representation of RBDs and virus binding regions on APN. a-c. Ribbon representation of RBDs from PDCoV, HCoV-229E and PRCoV. The receptor binding motifs (RBMs) on each RBD are colored in red. Compared to HCoV-229E and PRCoV, the RBD of PDCoV is shorter and include residues from the β-barrel core. **d-e**. Electrostatic potential distribution of hAPN, pAPN, HCoV-229E RBD and PDCoV RBD. Neutral residues, positively and negatively charged residues are colored in white, blue and red, respectively. The boundaries of PDCoV RBD binding regions on hAPN and pAPN are circled with green and cyan dotted lines, while the yellow dotted lines enclose the HCoV-229E RBD binding regions on hAPN. Accordingly, the RBMs on PDCoV RBD and HCoV-229E RBD are also enclosed with dotted lines.

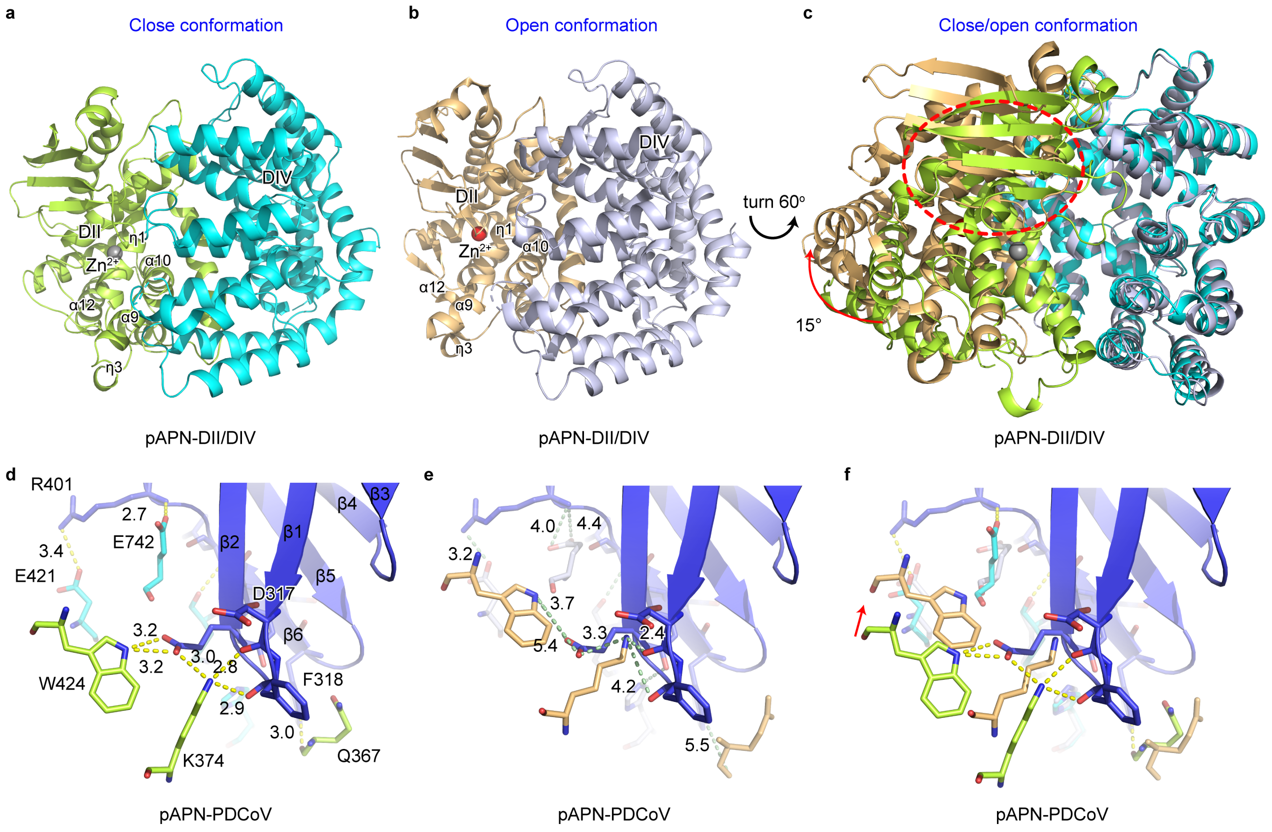

Supplementary Fig. 2 Open and close conformations of pAPN. a Ribbon representation of pAPN in close conformation. DII and DIV of pAPN are colored in green and cyan respectively, while the DI and DIII of pAPN are not shown for clarity. Zinc ion is located at the active site of the enzyme. **b** Ribbon representation of pAPN in open conformation. DII and DIV of pAPN are shown in orange and light blue, respectively. **c** Superposition of pAPN in two conformations as shown in **a** and **b**. DII swings about 15° toward DIV when pAPN changes from open to close conformation, closing the active site. **d** Zoom in view of the atomic interactions between PDCoV RBD and pAPN in close conformation. Residues on PDCoV RBD are colored in blue, whereas residues on DII and DIV of pAPN are colored in as in **a**. Oxygen and nitrogen atoms from each residue are shown in red and blue, respectively. Yellow dotted lines indicate the polar interactions (hydrogen bonds and salt bridges),

with the distance between interacting atoms labelled. **e** Docking of PDCoV RBD to pAPN in open conformation. Most of the interactions shown in **d** are now disrupted as a result of relative movement of DII and DIV. **f** Superposition of **d** and **e** to show the movement of key residues when pAPN switches conformations.

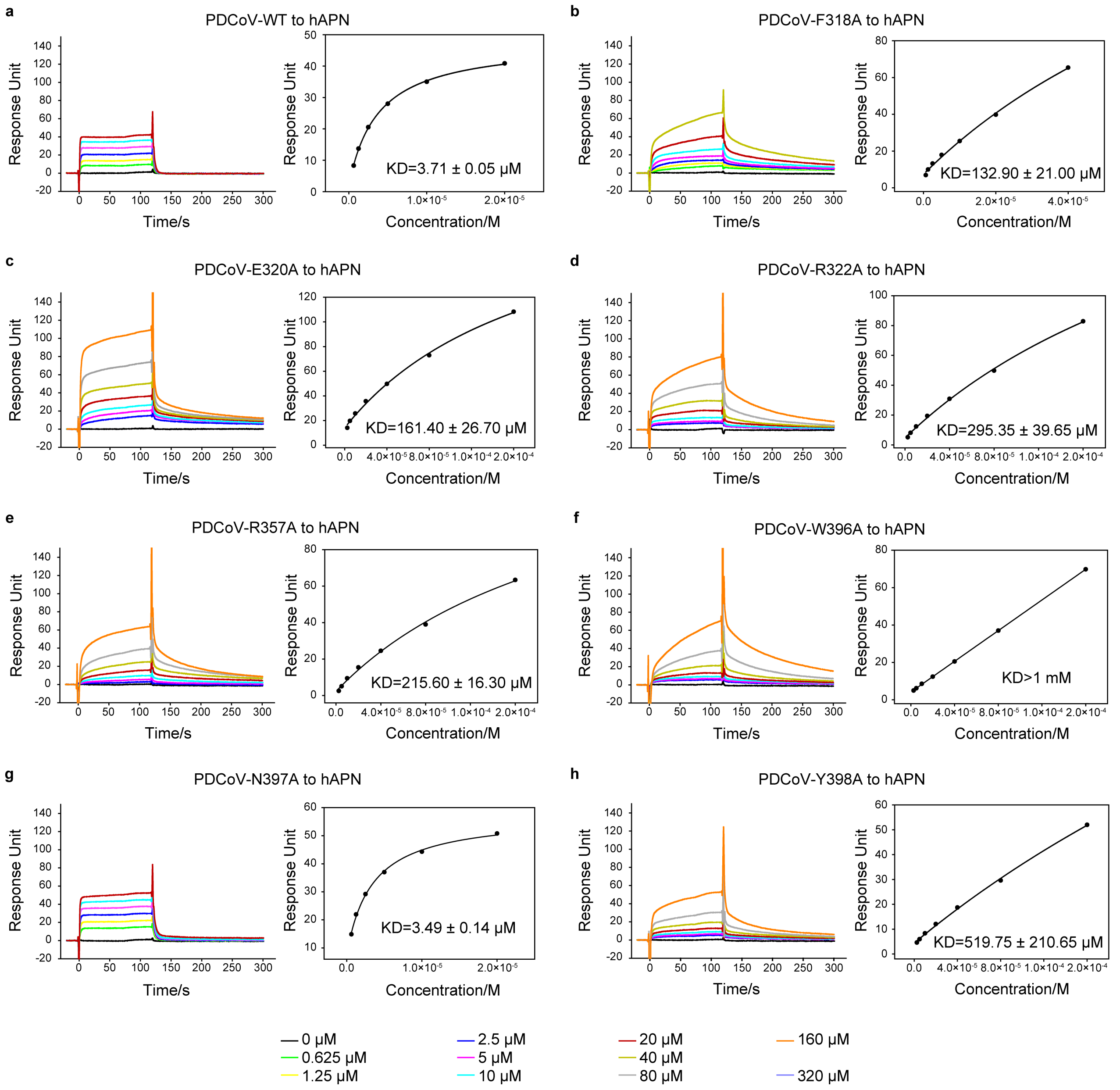

Supplementary Fig. 3 Binding of wildtype (WT) or mutant PDCoV RBD to hAPN measured by SPR. a Binding of PDCoV RBD WT to hAPN. **b-h** Binding of different PDCoV RBD mutants to hAPN. The equilibrium binding curve and derived dissociation constant is shown for each pair of interacting proteins. KD values are expressed as the mean ± SD, n=2. SPR sensograms are provided as a Source Data file.

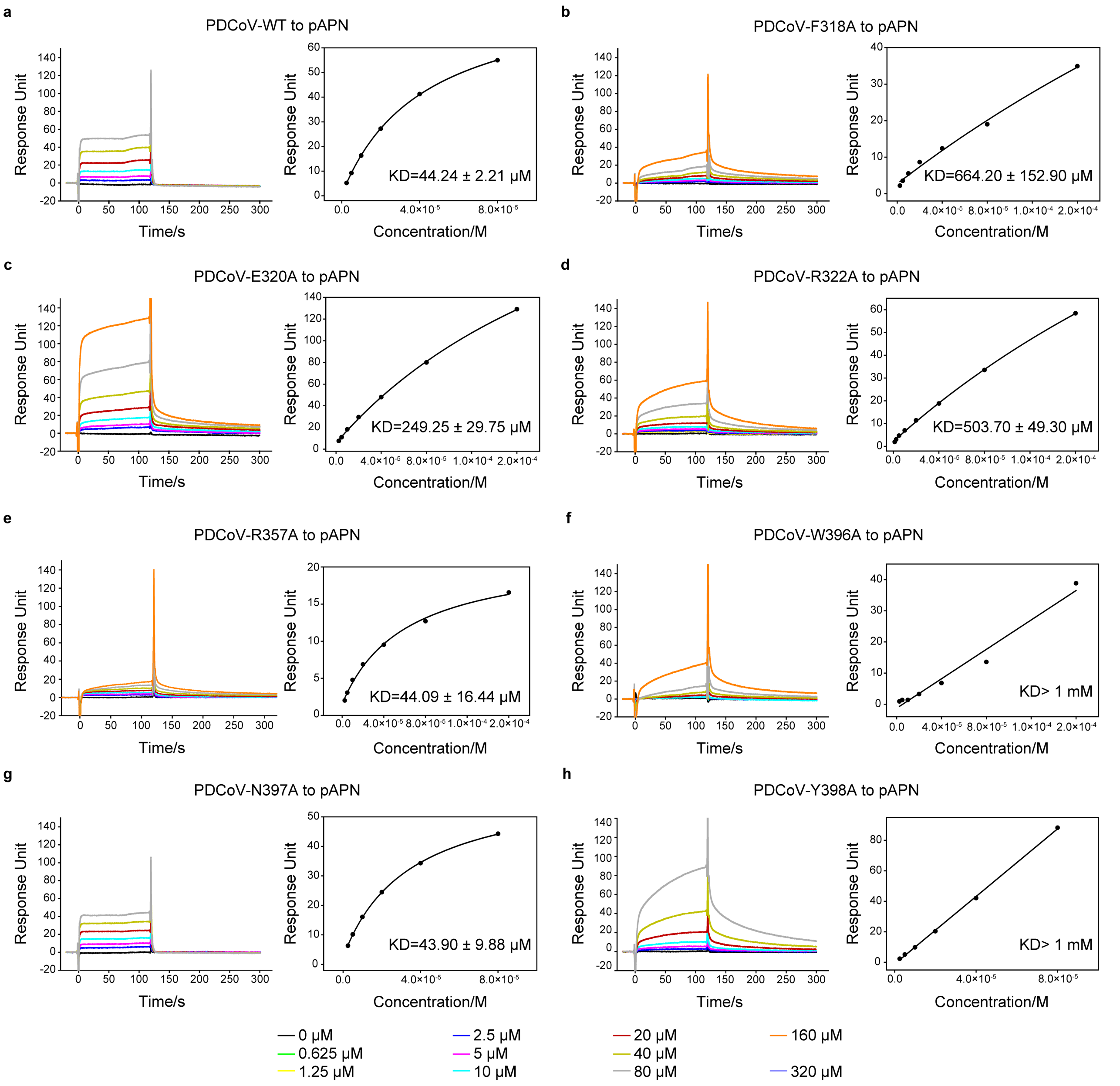

Supplementary Fig. 4 Binding of WT or mutant PDCoV RBD to pAPN measured by SPR. a Binding of PDCoV RBD WT to pAPN. **b-h** Binding of different PDCoV RBD mutants to pAPN. The equilibrium binding curve and derived dissociation constant is shown for each pair of interacting proteins. KD values are expressed as the mean ± SD, n=2. SPR sensograms are provided as a Source Data file.

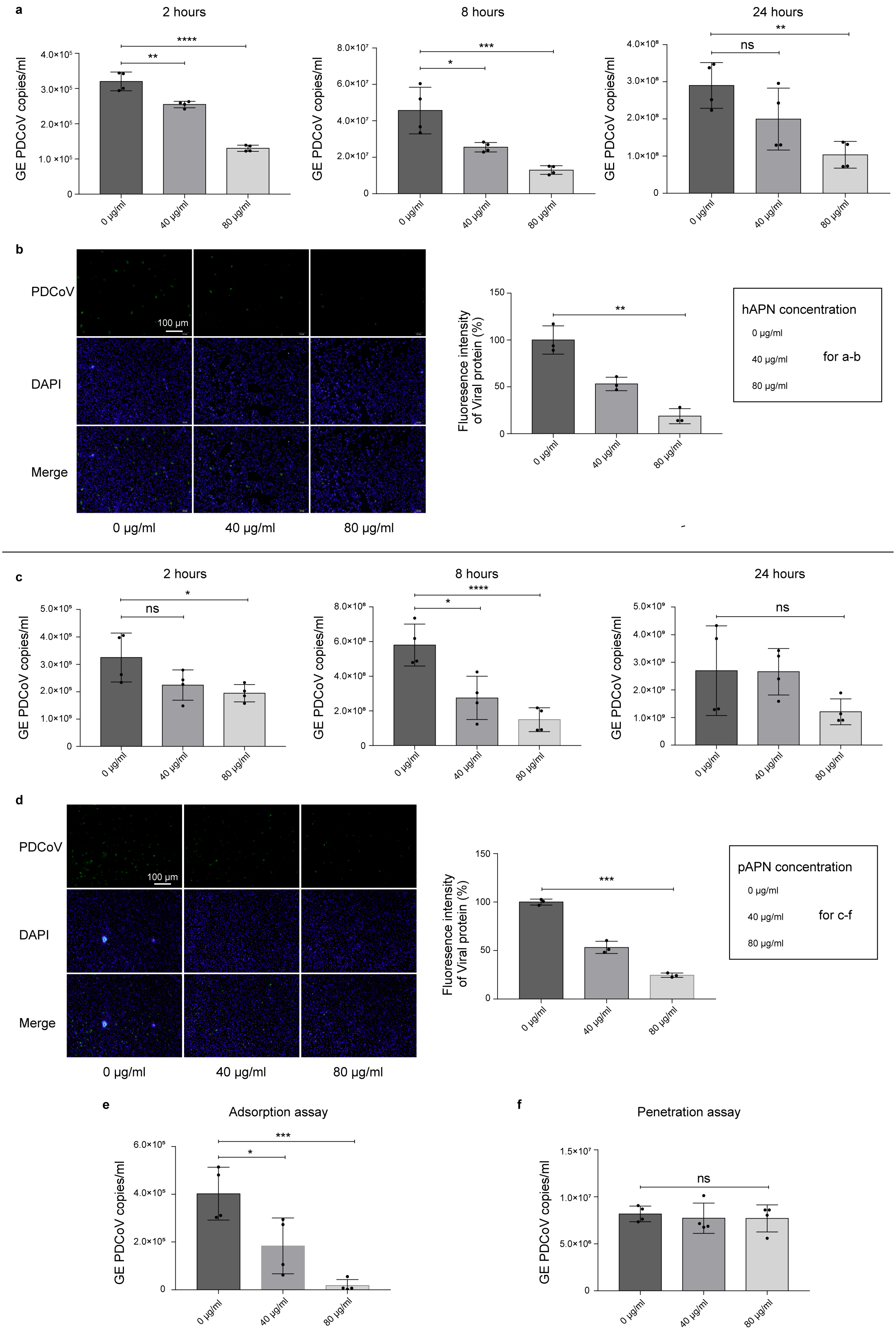
a
2 hours
8 hours
24 hours
GE PDCoV copies/ml
0 μg/ml
40 μg/ml
80 μg/ml
ns
b
PDCoV
DAPI
Merge
100 μm
0 μg/ml
40 μg/ml
80 μg/ml
Fluorescence intensity of Viral protein (%)
hAPN concentration
0 μg/ml
40 μg/ml
80 μg/ml
for a-b
c
2 hours
8 hours
24 hours
GE PDCoV copies/ml
d
PDCoV
DAPI
Merge
100 μm
Fluorescence intensity of Viral protein (%)
pAPN concentration
0 μg/ml
40 μg/ml
80 μg/ml
for c-f
e
Adsorption assay
f
Penetration assay
GE PDCoV copies/ml

Supplementary Fig. 5 hAPN and pAPN inhibits PDCoV replication in a dose dependent manner. a PDCoV (MOI=0.1) was preincubated with the indicated concentrations of hAPN at 37 °C for 1 hour, then the mixtures were added onto monolayers of LLC-PK1 cells in 24-well plates. After incubation at 4 °C for 1 hour, the plates were washed twice with DMEM containing 7.5 μg/ml trypsin and then cultured at 37 °C with 5% CO_2. At 2, 8, and 24 hpi, the samples were harvested for RT-qPCR to quantify PDCoV genome. Data are expressed as mean ± SD, n = 4. Error bars denote standard deviations for four biological replicates. Unpaired two-tailed t-test was used to determine the statistical significance. *, p<0.05; **, p<0.01; ***, p<0.001; ****, p<0.0001; ns, no significance. **b** At 2 hpi, the infected LLC-PK1 cells from (a) were fixed with 4% paraformaldehyde for IFA. PDCoV (green) was detected with porcine polyclonal antibodies, and cell nuclei were stained with DAPI (blue). The fluorescence intensity was determined by software ImageJ version 1.52i. Scale bar, 100 μm. Data are expressed as mean ± SD, n = 3. Error bars denote standard deviations for triplicate samples. Unpaired two-tailed t-test was used to determine the statistical significance. **, p<0.01. **c** PDCoV (MOI=0.1) was preincubated with the indicated concentrations of pAPN at 37 °C for 1 hour, then the mixtures were added onto monolayers of LLC-PK1 cells in 24-well plates. After incubation at 4 °C for 1 hour, the plates were washed twice with DMEM containing 7.5 μg/ml trypsin and then cultured at 37 °C with 5% CO_2. At 2, 8, and 24 hpi, the samples were harvested for RT-qPCR to quantify the PDCoV genome. Data are expressed as mean ± SD, n = 4. Error bars denote standard deviations for four biological replicates. Unpaired two-tailed t-test was used to determine the statistical significance. *, p<0.05; ****, p<0.0001; ns, no significance. **d** At 2 hpi, the infected LLC-

PK1 cells from (c) were also fixed with 4% paraformaldehyde for IFA. PDCoV (green) was detected with porcine polyclonal antibodies, and cell nuclei were stained with DAPI (blue). The fluorescence intensity was determined by software ImageJ version 1.52i. Scale bar, 100 μm. Data are expressed as mean ± SD, n = 3. Error bars denote standard deviations for triplicate samples. Unpaired two-tailed t-test was used to determine the statistical significance. ***, p<0.001. **e** LLC-PK1 cells in 24-well plates were incubated with 80 μg/ml pAPN for 1 hour at 37 °C, and then prechilled at 4 °C for 1 hour. The medium was then replaced with a mixture of pAPN (80 μg/ml) and PDCoV (MOI=0.1) in the presence of 7.5 μg/ml trypsin for 1 hour at 4 °C. After washing three times with prechilled PBS, PDCoV M protein gene levels were measured by RT-qPCR. Data are expressed as mean ± SD, n = 4. Error bars denote standard deviations for four biological replicates. Unpaired two-tailed t-test was used to determine the statistical significance. *, p<0.05; ***, p<0.001. **f** LLC-PK1 cells in 24-well plates were prechilled at 4 °C for 1 hour and then infected with PDCoV (MOI=0.1) for 1 hour at 4 °C. The virus-containing medium was replaced with fresh medium containing pAPN (80 μg/ml) in the presence of 7.5 μg/ml trypsin. The temperature was raised to 37 °C for 3 hours and then the cells were washed with PBS (pH 3) and collected for RT-qPCR. GE, genome equivalents. Data are expressed as mean ± SD, n = 4. Error bars denote standard deviations for four biological replicates. Unpaired two-tailed t-test was used to determine the statistical significance. ns, no significance. Values for fluorescence intensity and GE are provided as a Source Data file.

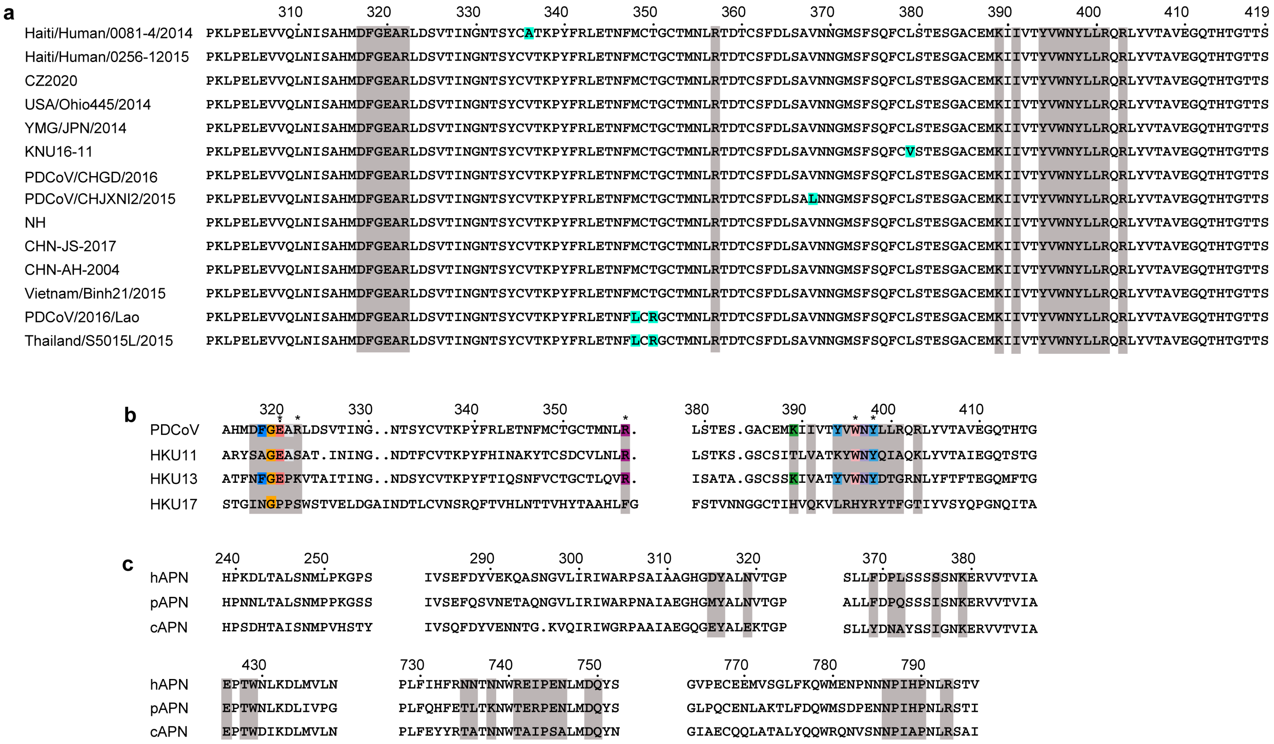

Supplementary Fig. 6 Comparisons of S protein from deltacoronaviruses and APN derived from different species. a Sequence alignment of RBD sequences of PDCoV isolated from different countries. RBMs are shaded grey, while non-identical residues are shaded cyan. **b** Sequence alignment of S protein from PDCoV, HKU11, HKU13 and HKU17. Shaded residues indicate RBMs on PDCoV RBD and counterparts from other deltacoronaviruses. Residues on PDCoV RBD essential for binding to hAPN or pAPN are marked with stars. Mutation of these residues dramatically decrease the affinity of PDCoV RBD to hAPN or pAPN. **c** Sequence alignment of hAPN, pAPN and chicken APN (cAPN). Shaded residues indicate virus binding motifs (VBMs) from hAPN and pAPN and corresponding residues from cAPN.

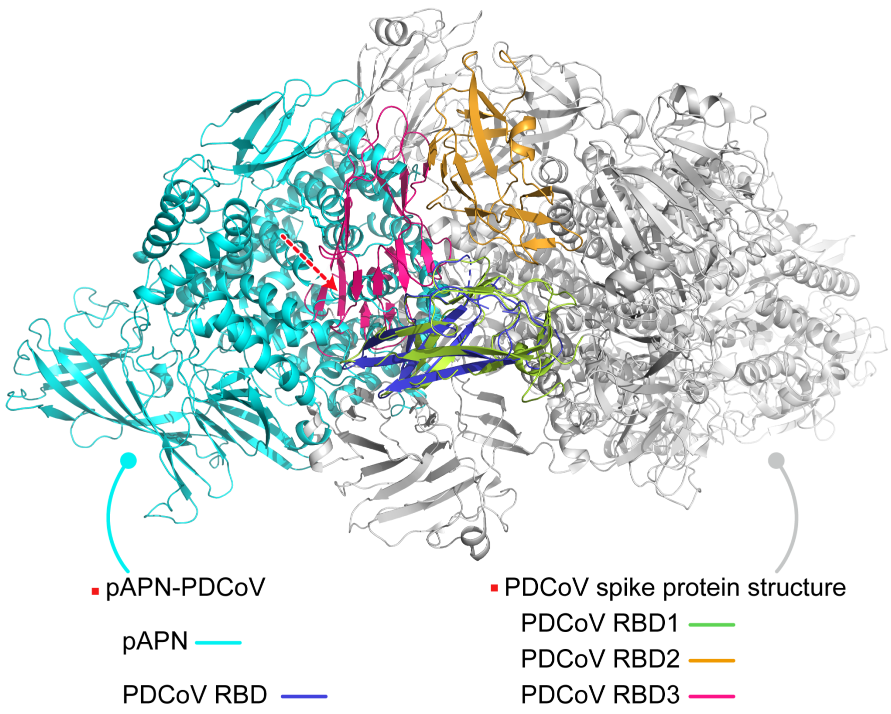

Supplementary Fig. 7 Superposition of the structures of PDCoV RBD-pAPN complex and PDCoV S protein ectodomain. pAPN and PDCoV RBD are colored in cyan and blue respectively. The three RBDs from PDCoV S protein trimeric structure are shown in green, orange and red, respectively, while the rest of the S protein trimer are colored in gray. Severe clashes, as indicated by dashed arrow, are generated between APN and S protein trimer when superpose two sets of PDCoV RBDs together.

Supplementary Table 1 List of contact residues between PDCoV RBD and hAPN / pAPN (residues on APN DIV are bolded)

PDCoV RBD	hAPN	pAPN
D317	K379	K374
F318	D315 Y316 N319<br>F369 P371 K379	M310 Y311 N314<br>F364 P366 K374
G319	P371	P366
E320	K379 E426 W429	K374 I371 E421<br>W424 **E742 Q747**
A321	**E745**	**E742**
R322	K379 E426 T428	E421 T423 W424
R357	**N736 N738 E742**	**E739**
K389	**N736**	**T732 L733**
I391	**E742**	**E739**
Y394	L372	Q367
V395	L372	Q367
W396	L372 **N786 P787**<br>**I788 H789 P790 R793**	Q367 **N783 P784**<br>**I785 H786 P787 R790**
N397	**R741 E742 I743**	**T738 E739 R740**
Y398	**I743 H789**	**R740 H786**
L399	**N736 E742 I743**<br>**P744 E745**	**L733 E739 R740**<br>**P741 E742**
L400	**E745**	**E742**
R401	**E745 N746 D749**	**E742 N743 D746**
R403	**N736**	**L733**

Supplementary Table 2 The affinities and residual standard deviation (SE) of PDCoV (WT) binding to pAPN/hAPN (mutant)

	**PDCoV-WT**	
	**KD(M)**	**SE**
hAPN-Y316A	3.571E-05	4.3E-06
	3.846E-05	5.2E-06
hAPN-E426A	3.054E-04	4.6E-05
	3.616E-04	4.9E-05
hAPN-W429A	1.578E-04	2.4E-05
	9.698E-05	1.1E-05
hAPN-K379A	3.712E-04	2.5E-05
	5.162E-04	6.9E-05
hAPN-E742A	1.427E-05	1.1E-06
	1.321E-05	1.1E-06
hAPN-E745A	3.128E-06	3.5E-07
	2.998E-06	2.6E-07
hAPN-H789A	6.469E-06	1.0E-06
	6.030E-06	8.6E-06
pAPN-E421A	1.027E-04	1.2E-05
	1.465E-04	1.4E-05
pAPN-W424A	1.133E-04	8.7E-06
	1.192E-04	8.3E-06
pAPN-K374A	2.516E-05	7.3E-06
	1.820E-07	1.5E-06

Supplementary Table 3 The affinities and residual standard deviation (SE) of pAPN/hAPN (WT) binding to PDCoV RBD (WT/mutant)

	**hAPN**		**pAPN**	
	**KD(M)**	**SE**	**KD (M)**	**SE**
PDCoV-WT	3.763E-06	2.5E-07	4.203E-05	9.9E-07
	3.662E-06	3.0E-07	4.645E-05	7.2E-07
PDCoV-F318A	1.119E-04	3.8E-05	8.171E-04	7.3E-04
	1.539E-04	9.9E-05	5.113E-04	4.4E-04
PDCoV-E320A	1.347E-04	2.6E-05	2.790E-04	3.0E-05
	1.881E-04	3.9E-05	2.195E-04	2.2E-05
PDCoV-R322A	2.557E-04	2.6E-05	4.544E-04	6.4E-05
	3.350E-04	6.8E-05	5.530E-04	9.1E-05
PDCoV-R357A	1.993E-04	5.7E-05	2.765E-05	2.7E-06
	2.319E-04	5.3E-05	6.052E-05	9.8E-06
PDCoV-W396A	2.339	26	45.85	3.7E+03
	0.088	0.61	44.27	3.2E+03
PDCoV-N397A	3.347E-06	4.6E-07	3.402E-05	1.3E-06
	3.625E-06	6.7E-07	5.378E-05	1.4E-05
PDCoV-Y398A	3.091E-04	7.8E-05	2.574	10
	7.304E-04	2.6E-04	1.112	31
