# Supplementary material for: Structures of a deltacoronavirus spike protein bound to porcine and human receptors
Source: Nat Commun. 2022 Mar 18;13:1467. doi: 10.1038/s41467-022-29062-5 (PMC8933513; doi:10.1038/s41467-022-29062-5)
Supplement: Supplementary file 3 — Reporting Summary [file 41467_2022_29062_MOESM3_ESM.pdf]

nature portfolio

Corresponding author(s): Ying Xu, Bin Li, Shuijun Zhang

Last updated by author(s): Feb 21, 2022

Reporting Summary

Nature Portfolio wishes to improve the reproducibility of the work that we publish. This form provides structure for consistency and transparency in reporting. For further information on Nature Portfolio policies, see our Editorial Policies and the Editorial Policy Checklist.

Statistics

For all statistical analyses, confirm that the following items are present in the figure legend, table legend, main text, or Methods section.

n/a	Confirmed	
☐	☒	The exact sample size (*n*) for each experimental group/condition, given as a discrete number and unit of measurement
☐	☒	A statement on whether measurements were taken from distinct samples or whether the same sample was measured repeatedly
☐	☒	The statistical test(s) used AND whether they are one- or two-sided<br>*Only common tests should be described solely by name; describe more complex techniques in the Methods section.*
☒	☐	A description of all covariates tested
☒	☐	A description of any assumptions or corrections, such as tests of normality and adjustment for multiple comparisons
☐	☒	A full description of the statistical parameters including central tendency (e.g. means) or other basic estimates (e.g. regression coefficient) AND variation (e.g. standard deviation) or associated estimates of uncertainty (e.g. confidence intervals)
☐	☒	For null hypothesis testing, the test statistic (e.g. *F*, *t*, *r*) with confidence intervals, effect sizes, degrees of freedom and *P* value noted<br>*Give P values as exact values whenever suitable.*
☒	☐	For Bayesian analysis, information on the choice of priors and Markov chain Monte Carlo settings
☒	☐	For hierarchical and complex designs, identification of the appropriate level for tests and full reporting of outcomes
☒	☐	Estimates of effect sizes (e.g. Cohen's *d*, Pearson's *r*), indicating how they were calculated

Our web collection on statistics for biologists contains articles on many of the points above.

Software and code

Policy information about availability of computer code

Data collection	Diffraction data were collected at the Shanghai Synchrotron Radiation Facility (SSRF) BL18U1. RT-qPCR data were collected with ABI 7500 real-time PCR system. The fluorescence intensity data were collected with software ImageJ Version 1.52i.
Data analysis	phenix.refine-1.19 ; COOT 0.9.5; PyMOL 2.3; SigmaPlot 14.0; Biacore X100 Evaluation software 2.0.1; ImageJ 1.52i; GraphPad Prism Version 7.00

For manuscripts utilizing custom algorithms or software that are central to the research but not yet described in published literature, software must be made available to editors and reviewers. We strongly encourage code deposition in a community repository (e.g. GitHub). See the Nature Portfolio guidelines for submitting code & software for further information.

Data

Policy information about availability of data

All manuscripts must include a data availability statement. This statement should provide the following information, where applicable:

- Accession codes, unique identifiers, or web links for publicly available datasets
- A description of any restrictions on data availability
- For clinical datasets or third party data, please ensure that the statement adheres to our policy

Coordinates and structure factors of PDCoV RBD-hAPN complex and PDCoV RBD-pAPN complex are available in the Protein Data Bank under accession codes 7VPQ and 7VPP, respectively. The PDCoV complete genome sequence is deposited in GenBank under accession number OK546242.

Field-specific reporting

Please select the one below that is the best fit for your research. If you are not sure, read the appropriate sections before making your selection.

☒ Life sciences ☐ Behavioural & social sciences ☐ Ecological, evolutionary & environmental sciences

For a reference copy of the document with all sections, see nature.com/documents/nr-reporting-summary-flat.pdf

Life sciences study design

All studies must disclose on these points even when the disclosure is negative.

Sample size	Sample size is based on effective size from prior publications. Unless otherwise stated, at least triplicate independent samples were used in the experiments.
Data exclusions	No data were excluded from the analyses.
Replication	Biochemical experiments were each replicated two or three times. Virus infection assays were repeated three times. All experiments performed in this study were successful.
Randomization	Sample allocation was random in our study.
Blinding	The investigators were blinded to group allocation during data collection and analysis in this study.

Reporting for specific materials, systems and methods

We require information from authors about some types of materials, experimental systems and methods used in many studies. Here, indicate whether each material, system or method listed is relevant to your study. If you are not sure if a list item applies to your research, read the appropriate section before selecting a response.

Materials & experimental systems

n/a	Involved in the study	
☐	☒	Antibodies
☐	☒	Eukaryotic cell lines
☒	☐	Palaeontology and archaeology
☒	☐	Animals and other organisms
☒	☐	Human research participants
☒	☐	Clinical data
☒	☐	Dual use research of concern

Methods

n/a	Involved in the study	
☒	☐	ChIP-seq
☒	☐	Flow cytometry
☒	☐	MRI-based neuroimaging

Antibodies

Antibodies used	Goat Anti-Pig IgG/FITC (Abcam, Cat: ab6911, lot: GR3226030-3) was used for immunofluoresence assays with a dilution of 1: 1000, Porcine anti-PDCoV antibody produced in our lab was used for immunofluoresence assays with a dilution of 1: 500. Anti-DYKDDDDK-Tag Mouse mAb (Abmart, Cat: M20008S, lot: 314375) and Anti-β-actin Mouse mAb (Proteintech, Cat: 66009-1-Ig, cloneNo.: 2D4H5, lot: 10004156) were used for Western blot with a dilution of 1:5000. HRP-conjugated Affinipure Goat Anti-Mouse IgG(H+L) (Proteintech, Cat: SA00001-1; lot: 20000374) used for Western blot with a dilution of 1:2000 .
Validation	Porcine anti-PDCoV antibody produced in our lab was validated by indirect immunofluoresence assays and ELISA experiments by different investigators in our laboratory. All the experiments demonstrated that the antibody can react with PDCoV CZ2020 (GenBank accession No. OK546242.1) infected cells. (Validation raw data can be required upon request).<br>Anti-β-actin Mouse mAb: https://www.ptgcn.com/products/Pan-Actin-Antibody-66009-1-Ig.htm.<br>Anti-DYKDDDDK-Tag Mouse mAb: http://www.ab-mart.com.cn/page.aspx?node=%2060%20&id=%20968.

Eukaryotic cell lines

Policy information about cell lines

Cell line source(s)	LLC-PK1 cell line (ATCC, CL-101) was purchased from the American Type Culture Collection. BHK-21 (GNHa10) and Huh7 (SCSP-526) cell lines were gifts from National Collection of Authenticated Cell Cultures, China. Hi5 (B85502) and sf9 (11496015) insect cells were bought from Thermo Fisher.
Authentication	Cells were not authenticated by us.

Mycoplasma contamination	All cell lines were tested negative for mycoplasma contamination.
Commonly misidentified lines<br>(See ICLAC register)	No misidentified cell lines were used.
